# Effects of Whey Protein Hydrolysate Ingestion on Serum Uric Acid Levels in Adult Men: A Randomized, Double‐Blind, Parallel‐Group, Placebo‐Controlled Study

**DOI:** 10.1002/fsn3.71150

**Published:** 2025-11-02

**Authors:** Yuuki Somoto, Ayana Okuno, Kouji Nomaguchi, Manabu Nakano, Miyuki Tanaka, Izumi Kakiuchi, Masahiko Nakamura, Naoki Sakane

**Affiliations:** ^1^ Morinaga Milk Industry co., Ltd. Zama‐Shi Kanagawa Japan; ^2^ Matsumoto College of Nursing Nagano Japan; ^3^ Matsumoto City Hospital Nagano Japan; ^4^ National Hospital Organisation Kyoto Medical Center Kyoto Japan

**Keywords:** body mass index (BMI), estimated glomerular filtration rate (eGFR), renal function, uric acid, whey protein hydrolysate, xanthine oxidase

## Abstract

Elevated serum uric acid (SUA) levels are associated with several diseases. Whey protein hydrolysate (WPH: product name, MWPH) contains multiple bioactive peptides and exhibits xanthine oxidase inhibitory activity in vitro. This randomized, parallel group, placebo‐controlled study evaluates WPH supplementation on SUA levels in adult men with fasting SUA levels of 6.0–7.9 mg/dL. Participants consumed 5.0 g of WPH or a placebo daily for 12 weeks. The primary endpoint, SUA, was significantly reduced in the WPH group compared to the placebo group after 12 weeks (*p* = 0.004). Furthermore, WPH also contains peptides that inhibit fat accumulation in cells in vitro, and in this study, BMI was significantly reduced in the WPH group compared to the placebo group after 12 weeks (*p* = 0.002). In a healthy subgroup of participants not receiving drug therapy, WPH also improved serum creatinine (*p* = 0.009) and estimated glomerular filtration rate (*p* = 0.010), indicating potential renal benefits. No serious adverse events or side effects were observed throughout the trial. The findings indicate that WPH supplementation can effectively lower SUA levels and improve renal function, offering a promising dietary intervention to manage hyperuricemia and associated metabolic conditions.

## Graphical abstract

Whey protein hydrolysate ingestion reduced uric acid levels after 12 weeks. Body mass index decreased with whey protein hydrolysate ingestion. Renal filtration rate improved with whey protein hydrolysate ingestion. No serious adverse effects due to whey protein hydrolysate ingestion were observed.

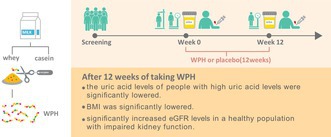

**Abbreviations**
BMI: body mass index
BW: body weight
Ccr: creatinine clearance
CUA: clearance of uric acid
eGFR: estimated glomerular filtration rate
EUA: urine uric acid excretion
ITT: intention‐to‐treat
PPS: per‐protocol set
SUA: serum uric acid
WPH: whey protein hydrolysate
XO: xanthine oxidase

## 1 Introduction

Elevated serum uric acid (SUA) levels can cause gout and are associated with obesity, diabetes, hypertension, cardiovascular disease, and chronic kidney disease (Kutzing and Firestein 2008; Raya‐Cano et al. 2022). The number of individuals with hyperuricemia worldwide is approximately 1 billion, although the definition of hyperuricemia slightly varies across countries. Its incidence varies with sex, age, and ethnicity. In Japan, the male‐to‐female ratio of hyperuricemia is approximately 10:1, with approximately 30% of adult males affected (Du et al. 2024). Although most patients are asymptomatic, SUA levels should be maintained < 6.0 mg/dL to reduce health risks (Richette et al. 2017).

Possible causes of hyperuricemia include genetic and environmental factors such as diet. Foods rich in purines, particularly proteins, such as red meat and fish, increase uric acid levels. In contrast, dairy products contain little purines (Kaneko et al. 2014; Kaneko et al. 2020) and make hyperuricemia less likely to develop through daily consumption. Epidemiological studies from Scotland, the United States, and Brazil have shown that intake of dairy products reduces SUA levels (Choi et al. 2004; Poletto et al. 2011; Zgaga et al. 2012). Casein and lactalbumin reduce uric acid levels more effectively than soy protein (Garrel et al. 1991). However, daily ingestion of a large amount of casein and lactalbumin (80 g/day) is difficult.

Whey protein hydrolysate (WPH) has demonstrated xanthine oxidase (XO) inhibitory activity in vitro and is expected to reduce SUA levels. Furthermore, WPH reduced adipocyte fat accumulation in vitro (Hirose et al. 2024).

This study aimed to investigate the effects of WPH (5.0 g/day) supplementation for 12 weeks on SUA levels in adult men with fasting SUA levels of 6.0–7.9 mg/dL in a randomized clinical trial. The estimated glomerular filtration rate (eGFR) was also evaluated.

## 2 Materials and Methods

### 2.1 Study Design and Ethical Approval

This randomized, parallel‐group, placebo‐controlled study was conducted at Matsumoto Junior College, Matsumoto City, Nagano Prefecture, Japan. This study was registered in the UMIN Clinical Trials Registry (UMIN000039465) and was approved by the Research Ethics Committee of Matsumoto Junior College (approval date: February 6, 2019, approval number: 201804). The study was conducted from December 2019 to July 2020 in accordance with the Declaration of Helsinki (Fortaleza, revised in October 2013) and the Ethical Guidelines for Medical Research Involving Human Subjects (Ministry of Education, Culture, Sports, Science and Technology; Ministry of Health, Labour and Welfare Notification No. 3, 2014). All participants provided written informed consent prior to participation.

### 2.2 Participants

The inclusion criteria were as follows: (1) male; (2) age 20–75 years; and (3) fasting SUA levels of 6.0–7.9 mg/dL at the time of the screening. The exclusion criteria were as follows: (1) regular intake of uric acid‐lowering food or supplements; (2) current treatment for hyperuricemia or gout; (3) history of gout, kidney stones, or rheumatoid arthritis; (4) history of serious or chronic illness involving the brain, liver, kidney, heart, lungs, digestive tract, or blood; (5) excessive alcohol consumption (> 60 g/day); (6) history of severe drug or food allergies; (7) participation in another clinical trial during the trial period or within 1 month before the date of obtaining consent; and (8) any condition deemed unsuitable for participation by the principal investigator based on the background of the participant, physical findings, and interview with the physician.

Participant characteristics were measured at weeks 0 and 12. Height was only measured in week 0. The body mass index (BMI) was calculated as weight divided by height squared (kg/m^2^).

### 2.3 Sample Size and Randomization

A sample size of 100 cases was calculated based on an estimated between‐group SUA difference of 0.4 mg/dL with a 0.7 standard deviation, significance level of 0.05, and power of 0.8. Assuming a dropout rate of 10% and an analysis exclusion rate of 10%, the target number of cases was set to 60 in each group for a total of 120 cases.

Participants were allocated in a 1:1 ratio using a stratified block randomization by a person in charge of allocation who was blinded to the test food. Allocation concealment was ensured by randomly assigning participants to test food numbers using computer‐generated random numbers. The allocation table was sealed and stored until data fixation upon completion of the study.

### 2.4 Intervention

The WPH was manufactured by Morinaga Milk Industry Co. Ltd. (MWPH; Tokyo, Japan), and contained 0.1% functional tetrapeptide Leu‐Asp‐Gln‐Trp (LDQW) as previously reported (Hirose et al. 2024). Active foods containing WPH (5.0 g) were purchased from Morinaga Milk Industry Co. Ltd. Dextrin was used as the placebo. Sweeteners, coloring agents, and flavoring were added to both products, which were then packaged into indistinguishable 8.7 g aluminum sticks. The two types of test food were confirmed to be indistinguishable by an independent researcher before and at the end of the study.

Participants consumed the active or placebo food once a day for 12 weeks. If the participant missed a dose, they were instructed not to consume it, but instead to record the missed doses in a diary. The remaining test food was collected and counted on the last day of week 12.

The participants were instructed to maintain their usual lifestyle and document daily food, alcohol, and supplements intake, as well as subjective symptoms. However, the planned 2‐week post‐observation period was canceled because of the COVID‐19 state of emergency.

Participants who did not consume at least 75% of the test food or began a new vigorous exercise routine during the study period were considered noncompliant.

### 2.5 Outcome Measurements

Blood and urine tests were initially scheduled for weeks 0, 4, 8, and 12; however, because of the COVID‐19 state of emergency, the tests were only performed at weeks 0 and 12. Blood tests included general biochemical tests and SUA levels. Urine samples were analyzed for specific gravity, creatinine, urine volume, and uric acid levels. The Matsumoto City Medical Association analyzed the serum and urine samples.

The primary outcome was the SUA levels. The secondary outcomes were different parameters measured according to the 2010 revised guidelines of the Japanese Society of Gout and Uric and Nucleic Acids. The clearance of uric acid (CUA) was calculated as follows: CUA = urinary urate × 60 min urine volume/(SUA × 60) × 1.73/body surface area. Creatinine clearance (Ccr) was calculated as follows: Ccr = urine volume × urine creatinine/(serum creatinine × min) × 1.73/body surface area. Creatinine excretion fractions were calculated as follows: (uric acid × serum creatinine)/(SUA × urine creatinine). Urinary urate excretion was calculated as follows: urinary urate × (urine volume/100)/body weight/hour. Classification type was grouped according to urinary urate excretion and CUA based on the 2010 revised guidelines of the Japanese Society of Gout and Uric and Nucleic Acids. The eGFR was calculated as follows: eGFR = 194 × creatinine (mg/dL)^−1.094^ × age (years) ^−0.287^.

### 2.6 Adverse Events

Adverse events were defined as any signs, symptoms, or diseases that occurred during the study, as well as abnormal laboratory results. General health was confirmed using diary entries. Abnormal changes in laboratory values were determined based on the results of the blood tests at weeks 0 and 12 according to the judgment criteria for adverse drug reactions and abnormal laboratory values in clinical trials with antimicrobial agents by the Japanese Society of Chemotherapy. The severity and causality of symptoms were evaluated according to the Japanese translation of the National Cancer Institute's Common Terminology Criteria for Adverse Events Version 4.0 by the Japan Clinical Oncology Group. The relationship between adverse events and test food was determined by the principal investigator.

### 2.7 Statistical Analysis

Efficacy analysis was defined according to the per‐protocol set (PPS). Participant characteristics were expressed as mean ± standard deviation. Food intake rate was evaluated using the Mann–Whitney U test. Primary analysis was performed using an analysis of covariance with baseline values for all participants. Student's paired t‐test was used for intragroup comparisons, while unpaired Student's t‐test evaluated baseline comparability. Fisher's exact test confirmed the balance between the active and placebo groups. The number of patients and frequency of adverse events and side effects, along with the incidence rates, were tabulated for each group. Bias in the classification of adverse events and side effects and the incidence rates was analyzed using Fisher's exact test. All statistical analyses without Fisher's exact tests were performed using SPSS Statistics version 29.0.0 (IBM SPSS Statistics). Fisher's exact test was performed using EZR version 1.4. Statistical significance was set at *p* < 0.05.

## 3 Results

### 3.1 Background Characteristics

**FIGURE 1:**
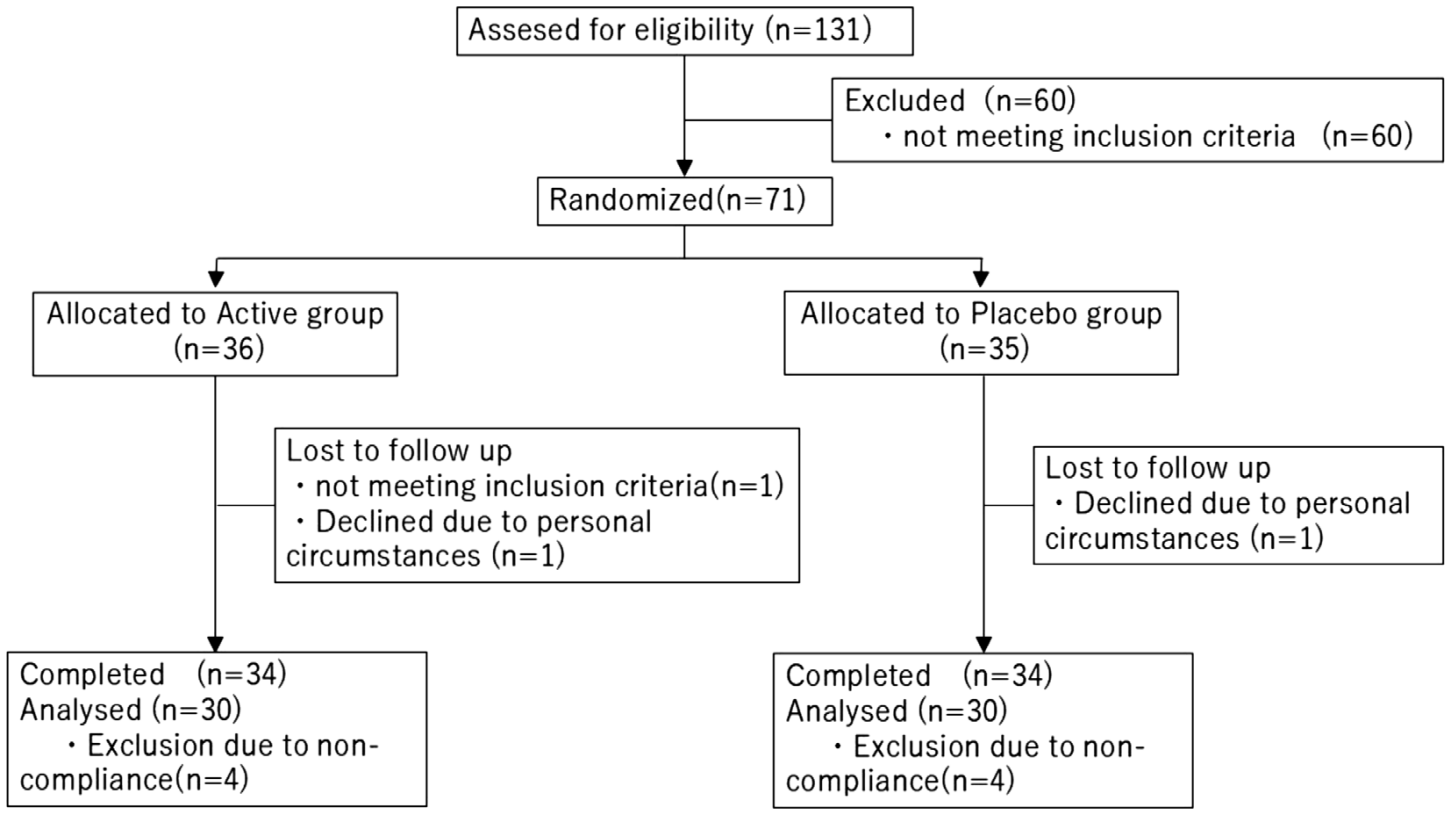
Participant flowchart.

Figure 1 shows the participant flowchart. Of the 131 participants who provided written consent, 71 were randomly assigned to either the active group (*n* = 36) or the placebo group (*n* = 35). After allocation, one participant in the active group failed to fulfill the inclusion criteria; thus, the intake period began with 35 participants in each group. The safety analysis consisted of 70 participants who consumed the test food at least once. During the intake period, one participant from each group withdrew; thus, a total of 68 participants completed the study (active group, *n* = 34; placebo group, *n* = 34). Five participants (active group, *n* = 3; placebo group, *n* = 2) had a consumption rate of < 75% and three participants (active group, *n* = 1; placebo group, *n* = 2) began intense exercise habits during the study; thus, eight participants were excluded for noncompliance. The PPS for efficacy analysis comprised 60 participants (*n* = 30 in each PPS group).

Eight participants who were taking medications for metabolic syndrome had no problems participating at the beginning of the study (active group, *n* = 5; placebo group, *n* = 3). A separate exploratory analysis was performed on a healthy subgroup, excluding those on medication (active group, *n* = 25; placebo group, *n* = 27). The background characteristics of this subgroup are shown in Table S1.

Table 1 shows the background characteristics of the intention‐to‐treat (ITT) and PPS groups. There were no significant differences in the baseline characteristics of the active and placebo groups. In contrast, the active group had significantly lower body weight and BMI than the placebo group at week 12 (body weight, *p* = 0.002, BMI, *p* = 0.002; Table 2). Body weight (*p* < 0.001) and BMI (*p* < 0.001) decreased significantly only in the active group after the intake period. Similarly, a significant difference was observed between the two groups in the healthy subgroup, and a decrease was observed only in the active group at week 12 (body weight, *p* < 0.001; BMI, *p* < 0.001; Table S2).

**TABLE 1:** Background characteristics of the ITT and PPS groups.

	ITT			PPS		
	Active	Placebo	*p*	Active	Placebo	*p*
Age (years)	52.58 ± 12.24	50.89 ± 12.10	0.559	54.03 ± 11.72	52.37 ± 11.81	0.585
Height (cm)	170.20 ± 4.54	171.27 ± 6.27	0.412	170.66 ± 4.40	170.91 ± 6.54	0.859
BW (kg)	71.35 ± 10.29	71.46 ± 9.89	0.111	71.55 ± 10.97	70.90 ± 9.20	0.804
BMI (kg/m^2^)	24.61 ± 3.19	24.31 ± 2.77	0.677	24.55 ± 3.43	24.24 ± 2.65	0.700
Alcohol consumption (g/day)^a^	12.71 ± 16.26	10.87 ± 13.43	0.611	12.53 ± 16.86	11.54 ± 14.13	0.808
SUA (mg/dL)	6.84 ± 0.54	6.85 ± 0.51	0.956	6.84 ± 0.53	6.88 ± 0.53	0.791
EUA (mg/kg/h)	0.45 ± 0.12	0.44 ± 0.10	0.444	0.45 ± 0.12	0.43 ± 0.09	0.479
CUA (mL/min)	7.33 ± 1.76	7.29 ± 1.69	0.915	7.40 ± 1.86	7.05 ± 1.57	0.428
Ccr (mL/min)	118.75 ± 22.04	117.58 ± 19.70	0.814	117.78 ± 23.60	117.56 ± 20.87	0.970
CUA/Ccr (%)	6.23 ± 1.30	6.25 ± 1.27	0.966	6.34 ± 1.31	6.03 ± 0.98	0.301
Serum creatinine (mg/dL)	0.96 ± 0.13	0.94 ± 0.12	0.620	0.95 ± 0.14	0.93 ± 0.12	0.501
eGFR (mL/min/1.73 m^2^)	67.63 ± 12.44	69.24 ± 11.19	0.570	67.45 ± 12.78	69.49 ± 11.37	0.516

*Note:* Data are presented as mean ± standard deviation, unless specified.

Abbreviations: BMI, body mass index; BW, body weight; Ccr, creatinine clearance; CUA, clearance of uric acid; eGFR, estimated glomerular filtration rate. EUA, urine uric acid excretion; ITT, intention‐to‐treat; PPS, per‐protocol set; SUA, serum uric acid.

^a^ ITT (active and placebo, *n* = 34) and PPS (active, *n* = 29) groups had missing values.

**TABLE 2:** Changes in the BW and BMI of the PPS group.

	Baseline	Week 12 (95% confidence interval)		*p*
		Active	Placebo	
BW (kg)	71.23 ± 1.30	69.89 ± 0.24 (69.42–70.37)	71.00 ± 0.24 (70.52–71.48)	0.002
BMI (kg/m^2^)	24.39 ± 0.39	23.95 ± 0.08 (23.78–24.12)	24.33 ± 0.08 (24.17–24.50)	0.002

*Note:* Data are presented as LS mean ± standard error, unless specified. Baseline refers to the average of all PPS participants.

Abbreviations: BMI, body mass index; BW, body weight; PPS, per‐protocol set.

We administered a dietary questionnaire assessing the meals eaten the day before the visits at weeks 0 and 12 and found no significant changes in meal content. Alcohol consumption increased in the ITT and PPS groups from weeks 0 to 12; however, there were no significant differences in alcohol consumption between the two groups at weeks 0 (*p* = 0.808) and 12 (*p* = 0.221).

### 3.2 Outcomes

#### 3.2.1 SUA Level

Table 3 summarizes the SUA results. The active group showed a significant decrease in SUA levels compared with the placebo group at week 12 (*p* = 0.004). We also performed a stratified analysis for those with normal SUA levels (6.0–7.0 mg/dL), which showed that the active groups had a significant decrease in SUA levels compared to the placebo group at week 12 (*p* = 0.032). The active group also showed a significant decrease in SUA levels after the test period (mild + normal range, *p* < 0.001; normal range, *p* = 0.013).

**TABLE 3:** Changes in the serum uric acid level of the PPS group.

	Baseline	Week 12 (*n*, 95% confidence interval)		*p*
		Active	Placebo	
Mild + normal range (6.0–7.9)	6.86 ± 0.07	6.60 ± 0.09 (30, 6.43–6.77)	6.96 ± 0.09 (30, 6.79–7.14)	0.004
Normal range (6.0–7.0)	6.51 ± 0.07	6.26 ± 0.10 (19, 6.07–6.45)	6.56 ± 0.10 (18, 6.37–6.76)	0.032

*Note:* Data are presented as LS mean ± standard error, unless specified. Baseline refers to the average of all PPS participants in the range specified.

Abbreviation: PPS, per‐protocol set.

In the healthy subgroup, the active group showed significantly lower blood uric acid levels than the placebo group at week 12 (*p* = 0.014; Table S3). A significant decrease in SUA levels was observed after intake in the active group (*p* = 0.010). A significant decline in SUA levels in the active group was also observed in the healthy subgroup (*p* = 0.025). An exploratory analysis comparing the number of participants who achieved SUA levels ≤ 6.0 mg/dL at week 12 showed that seven participants in the active group and one participant in the placebo group reached this threshold. Fisher's exact test yielded a *p*‐value of 0.052; when restricted to the normal range, the *p*‐value was 0.042.

In the active group, there was no significant increase in the urine uric acid excretion (EUA; Table 4, Table S4). There were no significant differences between or within the groups for CUA, Ccr, and CUA/Ccr. Participants were phenotyped based on EUA and CUA values according to the relevant guidelines. None of the participants had a mixed‐type hyperuricemia at week 0. Apart from the two participants in the placebo group who had a normal type classification, all participants had a decreased type of urate or renal excretion. There were no significant differences in the classification type between the groups (*p* = 0.261). At week 12, four participants in the active group and one in the placebo group had converted to the normal type, but there was no statistical bias in the distribution of each type between the groups (*p* = 0.203).

**TABLE 4:** Parameter changes in the PPS group.

	Baseline	Week 12 (95% confidence interval)		*p*
		Active	Placebo	
EUA (mg/kg/h)^a^	0.44 ± 0.01	0.44 ± 0.02 (0.40–0.48)	0.44 ± 0.02 (0.40–0.48)	0.901
CUA (mL/min)^a^	7.23 ± 0.22	7.51 ± 0.36 (6.80–8.23)	7.07 ± 0.36 (6.36–7.79)	0.390
Ccr (mL/min)^a^	117.67 ± 2.85	121.60 ± 5.01 (111.57–131.64)	119.33 ± 5.01 (109.30–129.36)	0.749
CUA/Ccr (%)^a^	6.19 ± 0.15	6.20 ± 0.18 (5.84–6.56)	5.98 ± 0.18 (5.62–6.33)	0.385
Serum creatinine (mg/dL)	0.94 ± 0.02	0.91 ± 0.01 (0.89–0.93)	0.94 ± 0.01 (0.92–0.96)	0.066
eGFR (mL/min/1.73m^2^)	68.47 ± 1.55	70.52 ± 0.86 (68.80–72.25)	68.62 ± 0.86 (66.89–70.34)	0.124

*Note:* Data are presented as LS mean ± standard error, unless specified. Baseline refers to the average of all PPS participants.

Abbreviations: Ccr, creatinine clearance; CUA, clearance of uric acid; eGFR, estimated glomerular filtration rate. EUA, urine uric acid excretion; PPS, per‐protocol set.

^a^ Both groups (*n* = 29) had some missing values at week 12.

#### 3.2.2 Renal Function Markers

The active group had lower serum creatinine levels than the placebo group at week 12 (Table 4). Serum creatinine significantly decreased in the active group at week 12 compared with week 0 (*p* = 0.004). The eGFR did not differ significantly between the groups at week 12, but a significant increase was observed in the active group at week 12 compared with week 0 (*p* = 0.010). In the healthy subgroup, serum creatinine and eGFR significantly improved in the active group compared with the placebo group at week 12 (creatinine, *p* = 0.009, eGFR, *p* = 0.010; Table S4). However, serum creatinine and eGFR significantly improved only in the active group at week 12 compared with week 0 (creatinine, *p* = 0.001, eGFR, *p* = 0.003).

### 3.3 Safety

A total of 52 adverse events were reported through the diaries of the participants and the blood and urine tests during the study period. None of the adverse events were serious, and no causal relationship with the test foods was identified by the principal investigator. The incidence rate was *n* = 16/35 in both groups, with no significant between‐group difference (*p* = 1.0).

## 4 Discussion

We conducted a clinical trial to investigate the effects of continuous intake of a food product containing 5.0 g of WPH on SUA levels. The primary outcome, SUA level, was significantly lower in the active group than in the placebo group after 12 weeks of intake, which suggests that continuous WPH intake can reduce SUA levels in individuals with mild and normal SUA levels (6.0–7.9 mg/dL). The clinical judgment threshold for gout in Japan is 6.0 mg/dL, according to the 2018 revised guidelines of the Japanese Society of Gout and Uric and Nucleic Acids. The preventive medical threshold for renal impairment is also 6.0 mg/dL. After 12 weeks of WPH intake, seven individuals in the test food group achieved SUA levels 6.0 mg/dL or less. The effect size of WPH on uric acid levels was considered clinically significant.

Participants were requested to maintain a daily food diary before and after the test food intervention, and they confirmed that these diaries represented their typical meal plans. Therefore, we believe that the observed effect was attributable to WPH.

We examined whether the reduction of uric acid was specific to a urate excretion type by measuring EUA and CUA. At week 12, no significant differences were observed in the distribution type between the groups. Among the four participants in the active group who changed to the normal excretion type at week 12, three had decreased uric acid excretion and one had renal overload. Based on these results, WPH may be effective regardless of its type. Toda et al. reported that WPH contains the functional tetrapeptide LDQW, which inhibits XO (Toda et al. 2025). XO inhibition reduces SUA levels; therefore, the efficacy of WPH has no difference in terms of excretion type (Du et al. 2024).

We also observed that the serum creatinine levels decreased significantly while the eGFR increased significantly after 12 weeks in the active group. Although there were no significant differences in creatinine levels and eGFR between the PPS groups, there were significant differences between the groups in the healthy subgroup. Since the renal function markers of the active group in the healthy subgroup showed greater improvements, it suggests that both high uric acid levels and the presence of metabolic syndrome may increase renal burden. These findings align with reports related to renal function in patients with early metabolic syndrome and high SUA (Xu, et al. 2024).

Previous studies have examined the effects of dairy products on uric acid levels. Specific amino acid sequences in whey hydrolysates have demonstrated XO‐inhibitory activity and prevented uric acid elevation in rodent models (Qi et al. 2022; Qi et al. 2023; Xu, Gong, et al. 2024). Reports on the effects of hydrolyzed whey in reducing uric acid levels have been reported in rat models with hyperuricemia and renal dysfunction induced by sodium oxonate (Qi et al. 2021). Milk‐derived tryptophan‐containing dipeptides have demonstrated XO‐inhibitory properties in vitro, and while not as potent as allopurinol, which is a treatment for hyperuricamia, their origin as food products is likely to have fewer side effects (Nongonierma and Fitzgerald 2012).

Compared with uric acid levels, there are few reports on kidney function and dairy products. A review on chronic kidney disease and dairy products reported that five out of seven studies prevented the onset of chronic kidney disease and a rapid decline in eGFR, especially low‐fat dairy products, while the other two reports found no significant effect (Eslami and Shidfar 2018).

Oxidative stress caused by NAD (P)H oxidase activation and expression is linked to hyperuricemia, nephropathy, and metabolic syndromes (Roumeliotis et al. 2019). Whey hydrolysates have demonstrated antioxidant activity (Blanca Hernández‐Ledesma et al. 2005; Pihlanto 2006), suggesting that whey‐derived peptides can improve renal function through antioxidative effects. In addition to XO inhibition, it is possible that these peptides have anti‐obesity properties. Metabolic syndrome, hyperuricemia, and renal dysfunction are interrelated, and the anti‐obesity effects of WPH may help alleviate hyperuricemia and renal dysfunction. A previous in vitro study showed that WPH contains the LDQW peptide, which inhibits adipocyte differentiation and upregulates genes that are involved in lipid metabolism (Hirose et al. 2024). The observed weight loss in this study likely reflects a reduction in body fat.

No serious adverse events were associated with WPH intake (5 g/day) during the study. In a previous review, the safety of whey and whey peptides was shown when these were used in large amounts for infant formulas and by athletes without any toxicity concerns (Zhao and Ashaolu 2020). These results support the safety of daily intake.

This study has several limitations. This study only included male participants, reflecting the higher incidence of hyperuricemia among men in Japan. However, the incidence of kidney damage varies less between men and women, with a ratio of 2:1 (Shiraishi et al. 2023). Because the COVID‐19 state of emergency was issued, observations could not be made at weeks 4 and 8. As a result, we were not able to track the improvements in BMI, SUA, and eGFR during the 12‐week period. Furthermore, continuous dietary records were not collected during the study period. Understanding the relationships between these factors can help elucidate the underlying mechanisms of WPH. The recruitment period was during the early stages of the COVID‐19 pandemic. Although the sample size was set to 120, only 71 participants met the inclusion criteria. Despite this, the observed effects suggest that WPH may have greater potency than expected.

## 5 Conclusions

Ingestion of 5.0 g of WPH significantly reduced SUA levels and improved eGFR in individuals with slightly elevated blood uric acid levels. WPH also significantly reduced BMI. These findings indicate that WPH may be useful for addressing kidney dysfunction and obesity, regardless of uric acid levels.

## Author Contributions

**Yuuki Somoto:** formal analysis (lead), writing – original draft (lead), writing – review and editing (lead). **Ayana Okuno:** formal analysis (equal), investigation (lead), methodology (equal). **Kouji Nomaguchi:** formal analysis (equal), investigation (supporting), methodology (equal). **Manabu Nakano:** visualization (supporting), writing – review and editing (equal). **Miyuki Tanaka:** conceptualization (equal), investigation (equal), supervision (lead). **Izumi Kakiuchi:** conceptualization (equal), investigation (equal). **Masahiko Nakamura:** investigation (equal), supervision (equal). **Naoki Sakane:** project administration (equal), supervision (equal).

## Conflicts of Interest

**Y.S**., **A.O**., **K.N**., **M.N**., and **M.T**. are employed by Morinaga Milk Industry Co. Ltd.

## Supporting information


**Tables S1‐S4.** Supporting Information.

## Data Availability

Data available on request due to privacy/ethical restrictions.
