# Supplementary figures and images for: Effects of Whey Protein Hydrolysate Ingestion on Serum Uric Acid Levels in Adult Men: A Randomized, Double‐Blind, Parallel‐Group, Placebo‐Controlled Study
Source: Food Sci Nutr. 2025 Nov 2;13(11):e71150. doi: 10.1002/fsn3.71150 (PMC12580292; doi:10.1002/fsn3.71150)

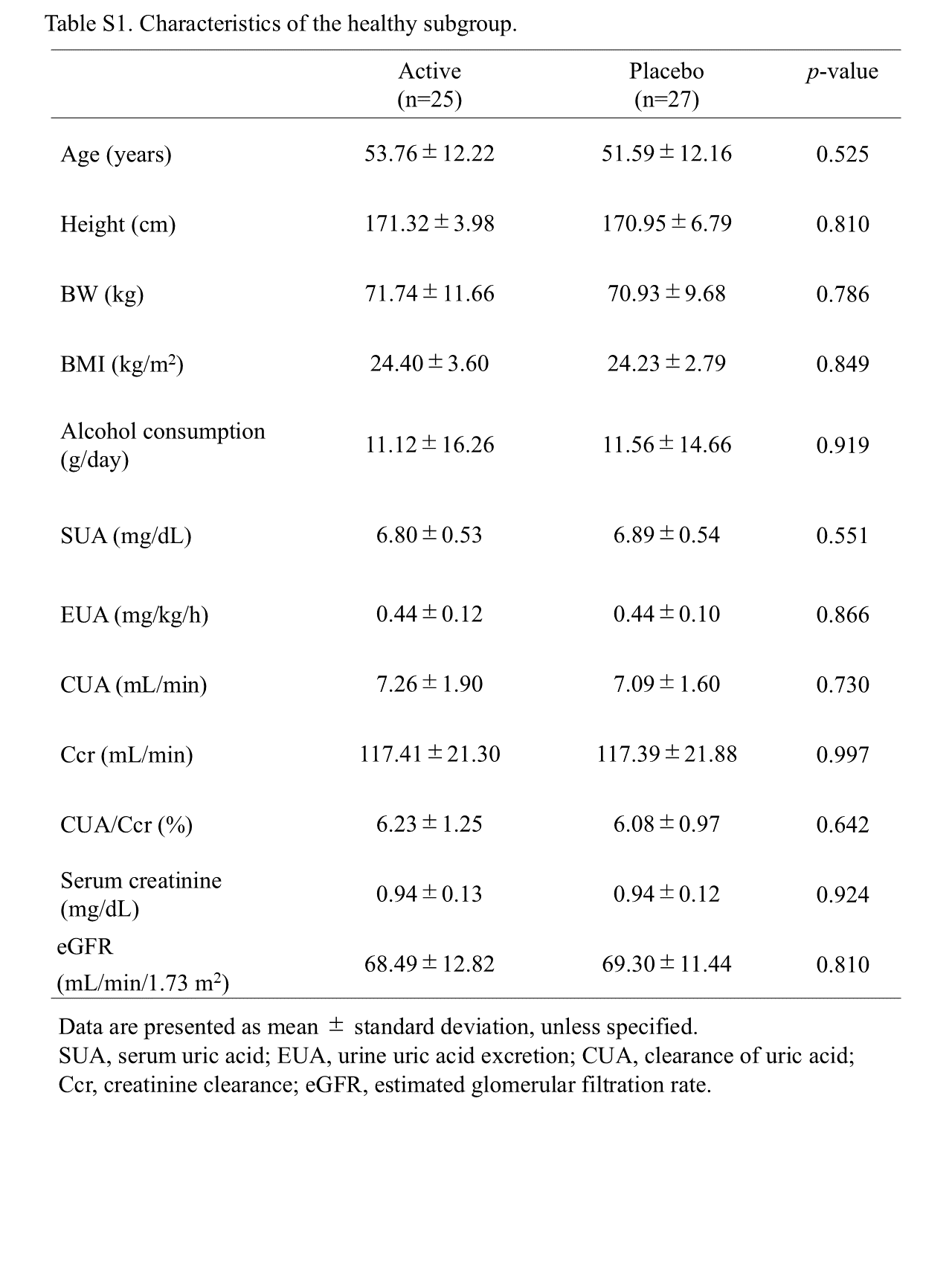


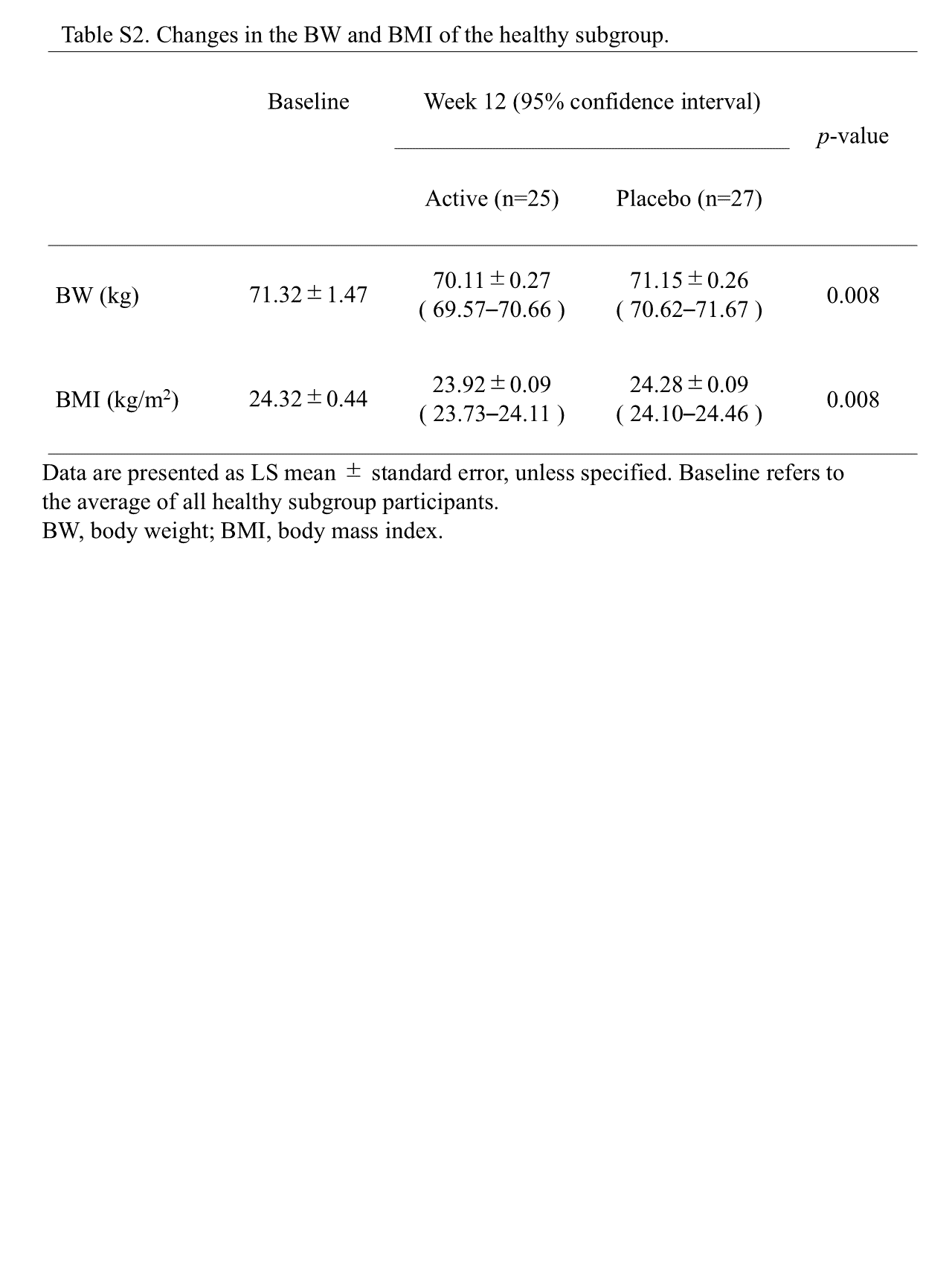


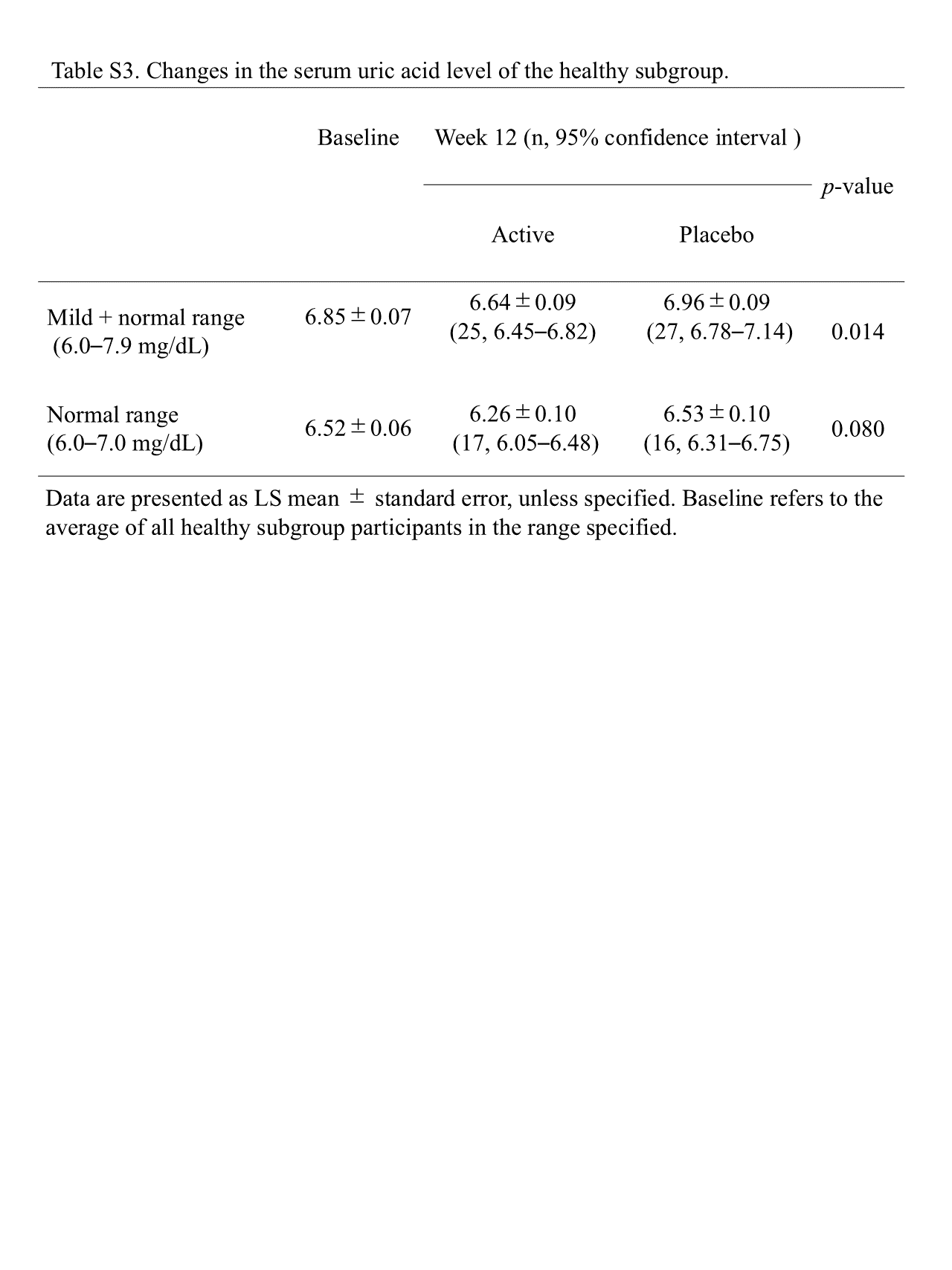


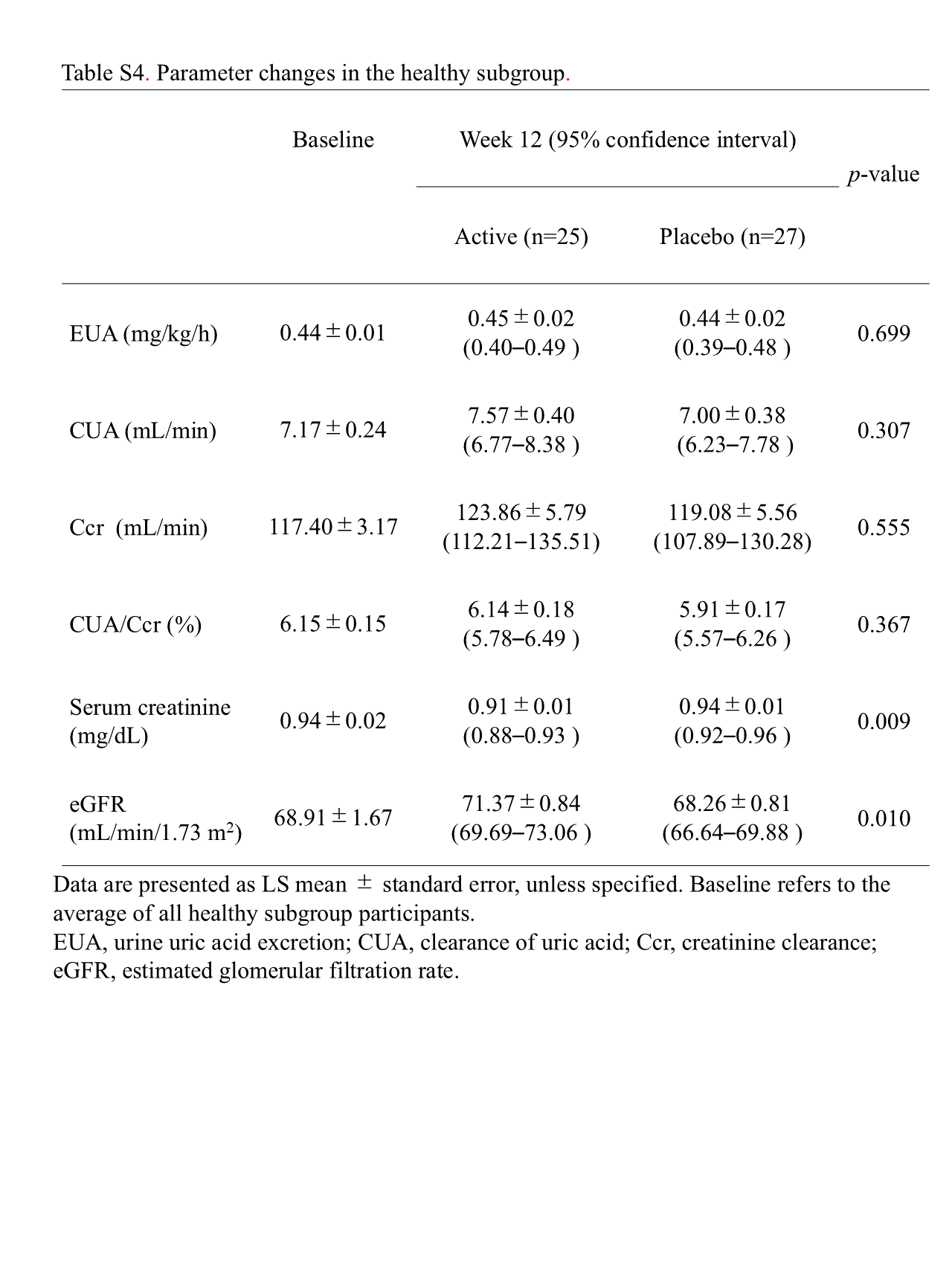

Supplement: Supplementary file 1 — Tables S1‐S4. Supporting Information. [file FSN3-13-e71150-s001.docx]
